# Shifting stoichiometry: Long‐term trends in stream‐dissolved organic matter reveal altered C:N ratios due to history of atmospheric acid deposition

**DOI:** 10.1111/gcb.15965

**Published:** 2021-11-05

**Authors:** Bianca M. Rodríguez‐Cardona, Adam S. Wymore, Alba Argerich, Rebecca T. Barnes, Susana Bernal, E. N. Jack Brookshire, Ashley A. Coble, Walter K. Dodds, Hannah M. Fazekas, Ashley M. Helton, Penny J. Johnes, Sherri L. Johnson, Jeremy B. Jones, Sujay S. Kaushal, Pirkko Kortelainen, Carla López‐Lloreda, Robert G. M. Spencer, William H. McDowell

**Affiliations:** ^1^ Department of Natural Resources and the Environment University of New Hampshire Durham New Hampshire USA; ^2^ Département des sciences biologiques Université du Québec à Montréal Montréal Québec Canada; ^3^ School of Natural Resources University of Missouri Columbia Missouri USA; ^4^ Environmental Studies Program Colorado College Colorado Springs Colorado USA; ^5^ Centre d’Estudis Avançats de Blanes (CEAB‐CSIC) Blanes Spain; ^6^ Department of Land Resources and Environmental Sciences Montana State University Bozeman Montana USA; ^7^ National Council for Air and Stream Improvement, Inc. Corvallis Oregon USA; ^8^ Division of Biology Kansas State University Manhattan Kansas USA; ^9^ Department of Natural Resources and the Environment, and the Center for Environmental Sciences and Engineering University of Connecticut Storrs Connecticut USA; ^10^ School of Geographical Sciences University of Bristol Bristol UK; ^11^ USDA Forest Service Pacific Northwest Research Station Corvallis Oregon USA; ^12^ Institute of Arctic Biology & Department of Biology and Wildlife University of Alaska Fairbanks Fairbanks Alaska USA; ^13^ Department of Geology University of Maryland College Park Maryland USA; ^14^ Finnish Environment Institute Helsinki Finland; ^15^ Department of Biological Sciences Virginia Polytechnic Institute and State University Blacksburg Virginia USA; ^16^ Department of Earth, Ocean and Atmospheric Sciences Florida State University Tallahassee Florida USA

**Keywords:** atmospheric acid deposition, C:N stoichiometry, dissolved organic carbon, dissolved organic matter, dissolved organic nitrogen, long‐term trends, streams

## Abstract

Dissolved organic carbon (DOC) and nitrogen (DON) are important energy and nutrient sources for aquatic ecosystems. In many northern temperate, freshwater systems DOC has increased in the past 50 years. Less is known about how changes in DOC may vary across latitudes, and whether changes in DON track those of DOC. Here, we present long‐term DOC and DON data from 74 streams distributed across seven sites in biomes ranging from the tropics to northern boreal forests with varying histories of atmospheric acid deposition. For each stream, we examined the temporal trends of DOC and DON concentrations and DOC:DON molar ratios. While some sites displayed consistent positive or negative trends in stream DOC and DON concentrations, changes in direction or magnitude were inconsistent at regional or local scales. DON trends did not always track those of DOC, though DOC:DON ratios increased over time for ~30% of streams. Our results indicate that the dissolved organic matter (DOM) pool is experiencing fundamental changes due to the recovery from atmospheric acid deposition. Changes in DOC:DON stoichiometry point to a shifting energy‐nutrient balance in many aquatic ecosystems. Sustained changes in the character of DOM can have major implications for stream metabolism, biogeochemical processes, food webs, and drinking water quality (including disinfection by‐products). Understanding regional and global variation in DOC and DON concentrations is important for developing realistic models and watershed management protocols to effectively target mitigation efforts aimed at bringing DOM flux and nutrient enrichment under control.

## Graphical abstract

The recovery of streams to atmospheric acid deposition has led to the changes in dissolved organic carbon (DOC) and dissolved organic nitrogen (DON) concentrations that have altered the stoichiometric ratios (DOC:DON) of the dissolved organic matter (DOM) pool. Certain sites have shown increasing DOC:DON trends (enrichment of the C‐rich fraction of the DOM pool), declining DOC:DON trends (enrichment of the N‐rich fraction of the DOM pool), and/or no clear trends in DOC:DON ratios. The responses vary geographically suggesting that the controls of DOM trends are at the regional to local scale.

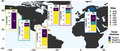

## 1 INTRODUCTION

Dissolved organic matter (DOM) provides an essential energy and nutrient source to aquatic ecosystems (Webster & Meyer, 1997). DOM varies in availability to biota along the hydrologic continuum (McArthur et al., 1985) and its composition and properties are closely linked to the surrounding landscape (Jaffé et al., 2008; Mattsson et al., 2005; Wymore et al., 2021c; Yates et al., 2019). The DOM pool is a complex mixture of organic compounds mostly composed of dissolved organic carbon (DOC) and dissolved organic nitrogen (DON) to a minor extent (Pagano et al., 2014). Numerous studies have confirmed that increases in DOC concentrations in north temperate freshwater ecosystems have occurred over time. For example, DOC concentrations have increased between 50% and 91% in streams and lakes of northern and central Europe, the United Kingdom, and eastern North America since the 1980s (Couture et al., 2012; de Wit et al., 2016; Driscoll et al., 2003; Evans et al., 2005; Gavin et al., 2018; Hall et al., 2021; Lawrence et al., 2011; Monteith et al., 2007; Worrall et al., 2004). Increased DOC concentration is often attributed to the recovery from acid deposition after the implementation of the Clean Air Act in the United States and similar legislation in Europe (Driscoll et al., 2003). The leading hypothesized mechanism is that a decrease in ionic strength and protonation in soil water following recovery from acid deposition leads to increases in solubility and the mobilization of DOC to adjacent water bodies (Borken et al., 2011; De Wit et al., 2007; Evans et al., 2005; Hruška et al., 2009; Lawrence & Roy, 2021).

A suite of different hypotheses has been put forward to explain the increasing trends in DOC concentrations, in addition to declines in atmospheric deposition, each associated with global change. Mechanisms include increasing precipitation and runoff (De Wit et al., 2007; Strååt et al., 2018; Worrall et al., 2004), rising CO_2_ and increased primary productivity (Freeman et al., 2004), enhanced microbial organic matter decomposition due to increased temperatures (De Wit et al., 2007; Finlay et al., 2006; Worrall et al., 2004), and permafrost thaw in northern high‐latitude ecosystems (Frey & McClelland, 2009; Frey et al., 2007; Frey & Smith, 2005; Larouche et al., 2015).

DOC concentrations are not increasing everywhere, however. Declines in DOC concentration over time have been associated with decreasing soil organic matter solubility (Clair et al., 2008), declines in carbon inputs from upstream acidified lakes (Schindler et al., 1997), increases in soil aluminum pools (Löfgren et al., 2010), and greater adsorption of DOM to the mineral layer and infiltration of DOM deeper into permafrost soils (Kendrick et al., 2018; Striegl et al., 2005). Long‐term stability in stream DOC concentrations has also been observed (Chow et al., 2017; Clair et al., 2008; Monteith et al., 2007; Worrall et al., 2004), even in some of the longest existing records of stream chemistry (e.g., since 1975; Räike et al., 2012). Trends in total organic carbon concentrations have even varied in direction within a continuous 35‐year record (Erlandsson et al., 2008; Lepistö et al., 2008). Despite the evidence that a wide range of changes in DOC concentration can be expected, a broad multi‐biome assessment of global DOC trends is lacking. A spatially distributed analysis would allow for the examination of trends along multiple environmental gradients and for the testing of coherent cross biome patterns (e.g., Dodds et al., 2019).

Concentrations of dissolved organic nitrogen (DON) are rarely measured in long‐term studies of DOM. Changes in DON concentration can have critical implications for freshwater ecosystems, especially when DON serves as a primary source of N for biota (Kissman et al., 2017; Mackay et al., 2020). While analytical challenges exist in the assessment of DON, researchers often assume that the concentrations of DON track those of DOC (i.e., concentrations are positively correlated; Campbell et al., 2000; Goodale et al., 2000; Kortelainen et al., 2006; Lepistö et al., 2008; Mann et al., 2012). Other lines of evidence, however, suggest that concentrations of DOC and DON can respond differently to environmental change such as changes in the concentrations of inorganic nutrients (Lutz et al., 2012; Wymore et al., 2015, 2021c; Yates et al., 2019) and seasonal variability in precipitation and stream runoff (Bernal et al., 2005). Recent evidence has pointed to the stream DOC:DON ratio varying according to the extent of nutrient enrichment in catchments, diverging from the soil DOC:DON ratio as systems become more nutrient‐enriched through land‐use change and increasing human population density (Yates et al., 2019). Such divergent trends in DOC and DON concentrations will lead to changes in DOM stoichiometry (i.e., DOC:DON ratios). DOC:DON ratios provide a relatively simple quantification of bulk DOM characteristics, which serves as an indicator of bioavailability (del Giorgio & Cole, 1998) and of changing OM sources within catchments (Yates et al., 2019). A broad assessment of how DOM stoichiometry changes concurrently with changes in concentrations of DOC and DON could provide insights into how the energy and nutrient balance of one of the larger pools of organic matter in freshwater ecosystems is changing with potential impacts on other biogeochemical reactions (e.g., Strauss & Lamberti, 2002; Wymore et al., 2019).

The objective of this study was to explore long‐term trends in DOC and DON concentrations, and DOM stoichiometry in streams and rivers across biomes of the Northern Hemisphere. Our overarching hypothesis is that changes in concentrations of DON will track those of DOC and consequently the stoichiometry of DOM will remain consistent through time (Brookshire et al., 2007; Wymore et al., 2021c). We also hypothesize that sites historically affected by acid deposition will be associated with increases in concentrations of DOC and DON assuming the same external forces are acting on each of these components of the DOM pool (Deininger et al., 2020). A global assessment of how riverine DOM is responding to global change is essential for robust regional and global scale predictive ecosystem models and for future watershed management protocols.

## 2 MATERIALS AND METHODS

### 2.1 Data set compilation

We compiled long‐term data on DOC and DON concentrations for 74 individual streams from 7 different sites (Table 1; Figure S1) in the Northern Hemisphere spanning 42 degrees of latitude (Tables S1 and S2). For each stream, DOC and DON data were collected at either weekly or monthly intervals, except for streams in a tallgrass prairie ecosystem (Konza Prairie: KNZ), for which we have limited DON data. For consistency across sites, we set minimum detection limits (MDL) for each solute: DOC (0.1 mg C/L), TDN (0.05 mg N/L), DON (0.01 mg N/L), NO_3_^−^ (0.005 mg NO_3_‐N/L), and NH_4_^+^ (0.004 mg NH_4_‐N/L). In addition, we only used DON values that were 5% or more of the TDN pool, to account for analytical uncertainty (Lloyd et al., 2016). For data points that were below the MDL, values were replaced with half the MDL. To estimate DOC and TDN from the Finnish data set, we multiplied TOC and TN by 0.95 (Kortelainen et al., 2006; Mattsson et al., 2005). These calculated TDN values for the Finnish data were then used to determine DON for these sites as: DON = TDN − (NO_3_^−^ + NH_4_^+^). Molar DOC:DON ratios were determined from the final DOC and DON concentrations and were used to describe change over time in the DOM pool. For more details on analytical methods see Table S4. Concentrations used in this study were not flow‐weighted as discharge data were not available for the same time frame as the chemistry time series nor available for all streams. Past work found that long‐term data collection can account for the variety of discharge values that occur at a site, and in at least one of our sites DOC concentrations were not correlated with discharge (Coble et al., 2018; Rüegg et al., 2015).

**TABLE 1:** Sites from which DOC, DON, and DOC:DON time series were obtained with the number of individual streams used from each site

Site	Site abbreviation	Biome	Acid deposition history	Individual streams	DOC (mg/L)	DON (mg/L)	DOC:DON Molar ratios	Geology	Soil type
Finland	FIN	Boreal forest	Yes	32	11.40 (1.9–78.85)	0.44 (0.02–12.40)	34.74 (20.89–74.47)	Granite	Ground moraine or silty clay
Caribou Poker Creek, AK	CPC^a^	Boreal forest	No	9	3.63 (0.27–29.44)	0.26 (0.02–1.91)	17.95 (10.98–19.78)	Quartz mica	Silt
Hubbard Brook Experimental Forest, NH	HBF^a^	Temperate deciduous forest	Yes	5	2.29 (0.18–24.49)	0.08 (0.01–0.42)	32.59 (31.37–63.53)	Granite	Sandy loam
Lamprey River Basin, NH	LMP	Temperate deciduous forest	Yes	9	4.80 (0.04–18.43)	0.19 (0.02–0.89)	30.79 (21.51–36.44)	Granite	Glacial till
H.J. Andrews Experimental Forest, OR	AND^a^	Coniferous temperate forest	No	9	0.99 (0.19–4.27)	0.03 (0.01–0.15)	34.61 (26.60–66.50)	Andesite and volcaniclastic	Sandy loam
Konza Prairie, KS	KNZ^a^	Tallgrass Prairie	Yes	2	0.88 (0.10–11.98)	–	–	Limestone	Silty clay
Luquillo Experimental Forest, PR	LUQ^a^	Tropical rainforest	No	8	1.05 (0.05–15.16)	0.05 (0.01–1.12)	23.67 (16.90–28.33)	Quartz diorite and volcaniclastic	Silty clay

Note

DOC, DON, and DOC:DON molar ratios median concentrations with minimum and maximum values, biome, geology, and soil type for each site.

‘–’ represents no data available.

^a^ Sites that are part of the long‐term ecological research network.

### 2.2 Time series and trend analyses

We examined time series from mean monthly DOC, DON, and DOC:DON values for each stream using the longest record possible from each site (Table S3) with the exception of the Arctic site, Caribou‐Poker Creeks Research Watersheds (CPC) where data are only available from May to August which coincides with the freshet and summer base flow periods. Time series were used to calculate trends using Sen slope (Hirsch et al., 1982) obtained from the *trend* package (Pohlert, 2018) in R (R Core Team, 2016) for DOC, DON, and DOC:DON ratios in each stream. Sen slope is a robust nonparametric method of regression, with the slope similar to the regression slope but less sensitive to outliers and reports a median change on the given parameter over time. Sen slopes with *p*‐value less than .05 were considered statistically significant indicators of either increasing or decreasing trends, while slopes with *p*‐values greater than .05 were considered insignificant and replaced with zeros for further analysis. The length of the data records across the 74 streams ranged from 8 to 45 years, where the longest starts in 1975 and all end between 2010 and 2015. Changes in analytical methods have been previously evaluated to ensure consistency over time (LUQ: McDowell et al., 2021; LMP: Coble et al., 2018; Wymore et al., 2021a; AND: Johnson et al., 2021; HBF: Campbell et al., 2021). Although we recognize that length of the record can be an important factor in trends over time (Argerich et al., 2013), we found no clear relationships between length of the record and trends in DOC, DON, and DOC:DON across our sites (Figure S2).

We used mutual information (MI) to determine the degree to which DOC and DON covary in each stream over time with the *muti* package (Scheuerell, 2017) in R (R Core Team, 2016). Mutual information is a non‐parametric method that characterizes the mutual dependence of two time series (Ardón et al., 2017; Cazelles, 2004). Here, we interpret that MI values closer to 1 indicate strong synchrony between DOC and DON where MI values closer to 0 indicate little dependency between the temporal dynamics of DOC and DON. Given that MI values do not provide information on the direction of the relationship, we paired MI values with eight categorical descriptions of the DOC and DON directional trends based on their respective Sen slopes. These categorical descriptions were: increasing DOC and DON, declining DOC and DON, increasing DOC with no trend in DON, increasing DOC with declining DON, no trend in DOC and increasing DON, no trends in DOC and decline in DON, a decline in DOC and no trend in DON and no trend in DOC and DON.

We obtained data from the National Atmospheric Deposition Program (NADP; NRSP‐3) to test the effect of atmospheric deposition on DOM trends for sites that have nearby NADP sampling locations (CPC, HBF, AND, KNZ, and LUQ). We used time series of NO_3_^−^ and SO_4_^2−^ fluxes to identify sites historically affected by atmospheric deposition (Figure S3). Sites showing a decline in atmospheric NO_3_^−^ and SO_4_^2−^ fluxes over time were classified as affected by atmospheric deposition and those that showed constant atmospheric NO_3_^−^ and SO_4_^2−^ fluxes were classified as sites that were not affected by acid deposition (Figure S3). We corroborated this approach with the expert knowledge of authors for their respective research sites. We used NADP data from HBF for LMP as these sites are in the same region. The sites across Finland were classified by their history in atmospheric acid deposition based on longitudinal patterns where southern Finland receives the greatest deposition (Ruoho‐Airola et al., 2014, 2015; Vuorenmaa, 2004).

We determined potential predictor variables of DOC, DON, and DOC:DON trends via an elastic net analysis, which is a form of penalized regression that shrinks variables that do not influence the model (Zou & Hastie, 2005). Elastic net produces a parsimonious model with the most influential variables and is minimally influenced by collinearity among predictor variables (Finlay et al., 2015). Lambda and alpha values were determined by cross‐validation and choosing the lowest mean squared error (Finlay et al., 2015). Lambda controls the shrinkage of variables while alpha selects the type of penalty where alpha values between 0 and 1 denote elastic net regression (Friedman et al., 2010). DOC, DON, and DOC:DON trends that were significant (*p* < .05) were included as the response variables into three different models focused on either ambient stream chemistry, watershed characteristics, or acid deposition history to determine if trends in DOM were related to in‐stream chemistry, the surrounding landscape or location, or atmospheric deposition history. The predictor variables for the ambient stream chemistry model were mean concentrations of DOC, DON, DOC:DON, NO_3_^−^, NH_4_^+^, Na^+^, and Ca^+2^ for each stream. Predictor variables for the watershed characteristics model were mean annual temperature (MAT, °C), mean annual precipitations (MAP, mm), mean watershed elevation (m), and watershed area (km^2^). The predictor variables for the atmospheric deposition model were mean and peak NO_3_^−^ and SO_4_^2−^ atmospheric deposition. We also tested categorical variables such as soil type (i.e., clay, silt, loam, till, and moraine), watershed geology (i.e., granitic, andesite, and volcaniclastic), and forest type (i.e., evergreen, conifer, deciduous, and mixed forest) with a Kruskal–Wallis rank sum test due to uneven sample size between groups with the *Stats* package (R‐Core‐Team, 2016). These categorical variables were not included in the models because they are poorly balanced and lead to the overfitting of models. Watershed characteristics were obtained from the site description on each LTER’s website and from Räike et al. (2012), Kortelainen et al. (2006), and Vuorenmaa (2004) for Finnish (FIN) sites. Atmospheric deposition values were obtained from the NAPD monitor site at the LTER sites, and models were fit using the *glmnet* package (Friedman et al., 2010) in R (R Core Team, 2016).

Differences in DOC, DON, and DOC:DON trends between acid deposition‐affected and unaffected sites were explored with a Kruskal–Wallis rank sum test due to uneven sample size between groups. We also conducted a one‐sample *t*‐test to determine if the means of DOM trends, in sites affected and not affected from acid deposition, were different from 0. This test is especially important for DOC:DON ratios to indicate whether changes in DOC and DON are proportional. The one‐sample t‐test was performed using the *Stats* package. All statistical analyses were conducted in R (R Core Team, 2016) using RStudio (version 1.2.1335, RStudio, Inc. Team, 2016).

## 3 RESULTS

### 3.1 DOC, DON, and DOC:DON trends

**FIGURE 1:**
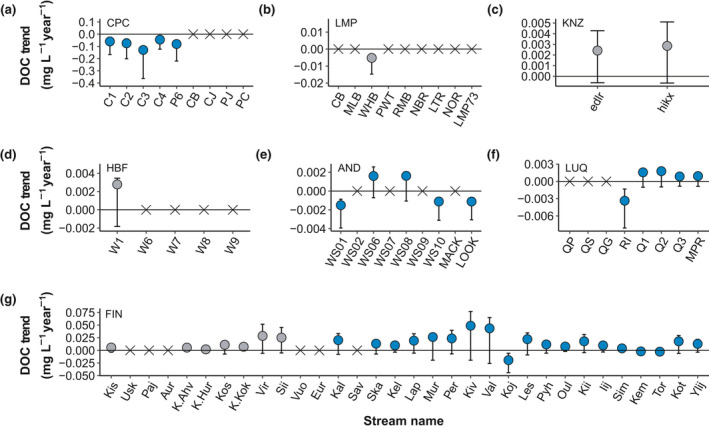
Sen slopes for DOC for each individual stream in (a) Caribou‐Poker Creeks Research Watershed (CPC), (b) Lamprey River Basin (LMP), (c) Konza Prairie (KNZ), (d) Hubbard Brook Experimental Forest (HBF), (e) H.J. Andrews Experimental Forest (AND), (f) Luquillo Experimental Forest (LUQ), and (g) Finland (FIN). Circles denote streams DOC trends (*p* < .05) along with their respective 95% confidence intervals and the X are streams with no DOC trends (*p* > .05). Blue are sites not affected by acid deposition and grey are sites historically affected by acid deposition. Caribou‐Poker Creeks, Lamprey, Hubbard Brook, and Andrews streams are ordered by watershed size from smallest to largest Luquillo streams are ordered by size within their respective major watersheds; Finland streams are ordered by latitude from South to North

**FIGURE 2:**
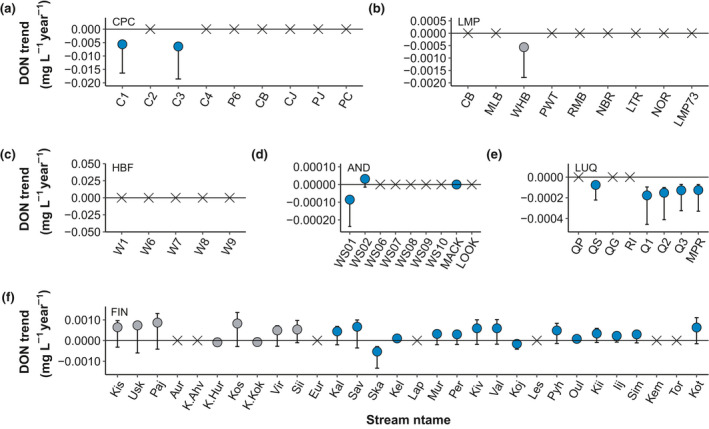
Sen slopes for DON for each individual stream in (a) Caribou‐Poker Creeks (CPC), (b) Lamprey River Basin (LMP), (c) Hubbard Brook Forest (HBF), (d) H.J. Andrews Forest (AND), (e) Luquillo Experimental Forest (LUQ), and (f) Finland (FIN). Circles denote streams with DON trends (*p* < .05) with their respective 95% confidence intervals and X denote streams with no trends (*p* > .05). Note Konza and 2 Finnish streams (YLIJOKI 1 and Vuoksi Vastuupuomi 061) were excluded due to no DON data available for these streams. Blue represents sites not affected by acid deposition and grey are sites historically affected by acid deposition. Caribou‐Poker Creeks, Lamprey, Hubbard Brook, and Andrews streams are ordered by watershed size; Luquillo streams are ordered by size within their respective major watersheds; Finland streams are ordered by latitude from South to North

Long‐term trends in concentrations of DOC (Figure 1) and DON (Figure 2) showed no consistent patterns across study sites. DOC trends ranged between −0.13 and 0.05 mg C/L per year (median = 0.003 mg C/L per year) while DON trends ranged from −0.006 to 0.0009 mg N/L per year (median = −0.0002 mg C/L per year). Trends for DOC:DON ratios ranged between −0.05 and 0.96 per year (median = 0.02 per year).

The majority of the streams had no significant temporal trend for either DON (36 of 70; 51%) or DOC:DON ratio (42 of 70; 60%; Figure 4). For DOC, a large portion of streams showed increasing trends (32 of 74; 43%), followed by 29 streams (37%) with no trends, and 13 streams (18%) with decreasing trends (Figure 4a). For DON, 21 (30%) and 13 (19%) of the streams had significant increasing and decreasing trends, respectively (Figure 4b). For DOC:DON ratios, 20 (29%) and 8 (11%) of the streams had significant increasing or decreasing trends, respectively (Figure 4c).

**FIGURE 3:**
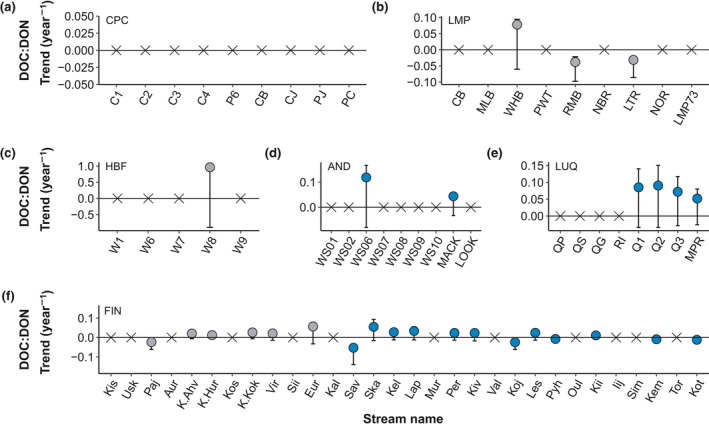
Sen slopes for DOC:DON molar ratios for each individual stream in (a) Caribou‐Poker Creeks (CPC), (b) Lamprey River Basin (LMP), (c) Hubbard Brook Forest (HBF), (d) H.J. Andrews Forest (AND), (e) Luquillo Experimental Forest LUQ), and (f) Finland (FIN). Circles denote streams with DOC:DON trends (*p* < .05) with their respective 95% confidence intervals and X denote streams with no trends (*p* > .05). Note Konza and 2 Finnish streams (YLIJOKI 1 and Vuoksi Vastuupuomi 061) were excluded due to no DON data available for these streams. Blue represents sites not affected by acid deposition and grey are sites historically affected by acid deposition. Caribou‐Poker Creeks, Lamprey, Hubbard Brook, and Andrews streams are ordered by watershed size; Luquillo streams are ordered by size within their respective major watersheds; Finland streams are ordered by latitude from South to North

The directionality of significant DOC trends was not consistent within the sites. At CPC, streams showed consistent decreasing DOC (Figure 1a) while KNZ and most of the FIN streams (44%) showed increases in DOC over time (Figure 1c,g). Other sites (LMP, AND, and LUQ; Figure 1b,e,f) exhibited variability in the direction of their significant DOC trends. Significant trends for DON were mostly declining across and within sites (Figure 2), except for FIN that had streams with both significantly increasing and declining DON trends (Figure 2f). DON trends were not significant at HBF (Figure 2c). Significant trends for DOC:DON ratios were generally positive (Figure 3) except for streams in LMP (Figure 3b) and FIN (Figure 3f), which showed declining stream DOC:DON ratios over time. There were no significant trends in DOC:DON ratios at CPC (Figure 3a).

### 3.2 Synchronicity between DOC and DON

Contrary to our hypothesis, trends in DON concentrations did not consistently track those of DOC. In only five of 68 streams (7%) did DON and DOC track each other, with MI values greater than 0.5. Of these five streams, in only three did DOC and DON covary in the same direction, both increasing or both declining in BNZ and FIN (Figure 5a,f). There were also various streams (18 out of 68) across sites where DOC and DON changed in a similar direction (either increased or decreased), but concentrations did not strongly covary according to MI values (i.e., MI < 0.5; Figure 5a,b,e,f). For 23 streams (33%), DOC and DON trends were asynchronous (i.e., DOC and DON changed in the opposite direction and MI < 0.5).

### 3.3 Acid deposition history

We did not find strong evidence to confirm our second hypothesis that DOC and DON would both increase in sites historically affected by atmospheric deposition. There was no difference in long‐term trends in DOC concentration (*p* = .38; Figure 6a) between sites historically affected by acid deposition (HBF, LMP, KNZ, and Southern FIN) and those not affected by acid deposition (CPC, AND, LUQ, and Northern FIN). The DOC Sen slope values for sites that were affected by acid deposition were different from zero (*t*‐test *p* = .008), whereas those unaffected by acid deposition were not different from zero (*t*‐test *p* = .63). Trends in DON differed with acid deposition history (*p* = .05, Figure 6b), with trends in acid deposition affected sites being greater than trends in sites not affected by acid deposition. The DON Sen slopes were different from zero for sites affected by atmospheric deposition (*t*‐test *p* = .05), but not for unaffected sites (*t*‐test *p* = .35). Trends in DOC:DON ratios did not differ in their response to acid deposition history (*p* = .56). DOC:DON Sens slopes for sites affected by acid deposition were not different from 0 (*t*‐test *p* = .33), whereas DOC:DON Sens slopes were significantly different from zero in sites unaffected by acid deposition (*t*‐test *p* = .008, Figure 6c).

### 3.4 Predictor variables of DOM trends

The elastic net models for chemistry and watershed characteristics identified several predictor variables for DOC, DON, and DOC:DON trends. For DOC trends, the ambient stream chemistry model accounted for a large percentage of variability, followed by acid deposition, and watershed characteristics: *r* ^2^ = .66, *r* ^2^ = .34, and *r* ^2^ = .32, respectively (Table 2). Variables selected for DOC trends were Ca^2+^ (*β* = −.005), Na^+^ (*β* = .002), and DOC (*β* = .002) in the stream chemistry model; MAT (*β* = .0006) and elevation (*β* = −.0001) for watershed characteristics; mean (*β* = −.004) and peak (*β* = .002) SO_4_^2−^ and peak NO_3_^−^ (*β* = −.0007) deposition. DOC trends did not vary across different geology types (Figure S4a), but DOC trends were the greatest in streams draining moraine and clay soil types (Figure S5a) as well as conifer forests (Figure S6a).

**TABLE 2:** Results of elastic net models exploring the influence of ambient stream chemistry (DOC, DON, DOC:DON, NO_3_^−^, NH_4_^+^, Ca^2+^, Na^+^), watershed characteristics (mean annual temperature (MAT °C), mean annual precipitations (MAP, mm), mean watershed elevation, and watershed area (km^2^)), and atmospheric acid deposition (mean and peak NO_3_^−^ and SO_4_^2−^ deposition (kg/ha)) on DOC, DON, and DOC:DON trends (for streams with significant Sen slopes) that were considered as response variables

DOC						
Model parameters	Stream chemistry		Watershed characteristics		Acid deposition	
*r* ^2^	.66		.32		.34	
*n*	43		41		39	
Lambda	0.003		0.0002		0.0001	
Alpha	.96		.96		.96	
	Variable	*β*	Variable	*β*	Variable	*β*
	Ca^2+^	−.005	MAT (°C)	.0006	Mean SO_4_^2−^	−.004
	Na^+^	.002	Elev. (m)	−.0001	Peak SO_4_^2−^	.002
	DOC	.002			Peak NO_3_^−^	−.0007

DON						
Model parameters	Stream chemistry		Watershed characteristics		Acid deposition	
*r* ^2^	.27				.34	
*n*	34				34	
Lambda	0.0004				0.00001	
Alpha	.21				.83	
	Variable	*β*			Variable	*β*
	DON	.001			Mean SO_4_^2−^	−.0004
	NO_3_^−^	.0002			Peak SO_4_^2−^	.0003
	Na^+^	.0001				
	Ca^2+^	−.0001				

DOC:DON						
Model parameters	Stream chemistry		Watershed characteristics		Acid deposition	
*r* ^2^	.10		.18		.01	
*n*	28		26		28	
Lambda	0.05		0.07		0.06	
Alpha	.58		.19		.94	
	Variable	*β*	Variable	*β*	Variable	*β*
	DON	−.10	Elev. (m)	.0002	Peak NO_3_^−^	.0007
	Ca^2+^	−.004				

Note

Lambda controls the shrinkage of variables while alpha selects the type of penalty where alpha values between 0 and 1 denote elastic net regression, and beta values are the model coefficients for the selected variables. KNZ was excluded for these models due to no DON data. No variables were selected in the DON trends watershed characteristics model.

For DON, the ambient stream chemistry and the acid deposition models explained 27% and 34% of the variability, respectively, in trends while the watershed characteristics model did not select any variables (Table 2). The highest *β* coefficients for the chemistry model were mean concentrations of DON (*β* = .001), NO_3_^−^ (*β* = .0002), Na^+^ (*β* = .0001), and Ca^2+^ (*β* = −.0001). For the acid deposition model, the variables selected were mean and peak SO_4_^2−^ deposition (*β* = −.0004 and .0003, respectively). Significant DON trends were only found in volcaniclastic and granitic watersheds, but trends were more constrained in volcaniclastic areas (Figure S4b). Similar to DOC trends, DON trends were the greatest in streams draining moraine and clay soil types and mostly negative for loam, silt, and sandy watersheds (Figure S5b) as well as greater in conifer forests (Figure S6b).

Lastly, the goodness of fit of the models for DOC:DON ratios was low with the stream chemistry and watershed characteristics models explaining 10% and 18% of the variability in trends, respectively. The acid deposition model only explained 1% of the variance (Table 2). The predictor variables for DOC:DON trends in the stream chemistry model were DON (*β* = −.10), and Ca^2+^ (*β* = −.004), elevation (*β* = .0002) in the watershed characteristics, and peak NO_3_^−^ deposition (*β* = .0007) for the acid deposition model. See Figures S4–S6 for more details on watershed characteristic relationships. Opposite to DOC and DON trends, DOC:DON trends were mostly positive in volcaniclastic watersheds and showed greater variability in the granitic sites (Figure S4c). There were no statistical differences in DOC:DON trends across soil types (Figure S5c) and differences across forest types were minimal except comparing DOC:DON trends between evergreen and conifer forests (Figure S6c).

## 4 DISCUSSION

**FIGURE 4:**
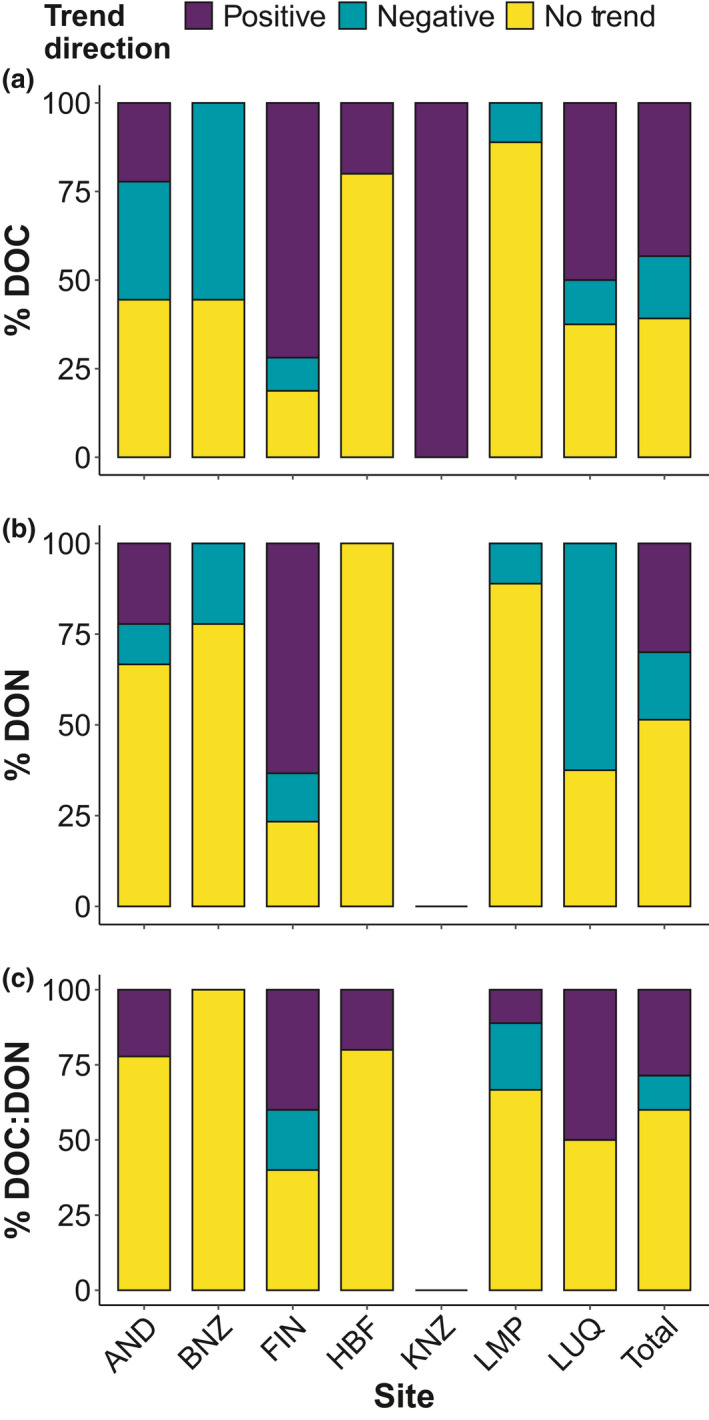
Percent of (a) DOC, (b) DON, and (c) DOC:DON ratios trends with positive (purple), negative (blue), or no trend (yellow) per site based on Sen slope *p* < .05. Note KNZ and 2 Finnish streams (YLIJOKI 1 and Vuoksi Vastuupuomi 061) were excluded from (b) and (c) due to no DON data available

This is one of the first comprehensive studies in which time‐series trends have been simultaneously determined for concentrations of DOC and DON and for DOM stoichiometry of the dissolved organic pool. We captured the inherent heterogeneity of DOM across a wide range of streams (74 individual streams) and six biomes by quantifying changes in three different metrics of the ambient DOM pool. Contrary to expectations from earlier studies exploring increases in DOC concentrations in freshwater systems, primarily in temperate New England, the United Kingdom, and parts of Europe, concentrations of DOC and DON did not vary over time in any consistent pattern across study sites. Although we found 43% of our streams increasing in DOC (Figure 4), the majority of sites exhibited no significant long‐term trends (Figure 4; Arvola et al., 2004; Clair et al., 2008; Coble et al., 2018; Räike et al., 2012; Rodríguez‐Murillo et al., 2015), suggesting that increasing DOC is not ubiquitous across the landscape and that local context influences these long‐term trends. Increasing DOC concentrations were also not exclusive to sites affected by acid deposition. For example, streams in the tropical rainforest site (LUQ) exhibit mostly positive trends in DOC concentrations that could be related to the high frequency of storm events (Wymore et al., 2017) that can also lead to an increase in the decomposition of organic matter (McDowell et al., 2013) rather than atmospheric deposition history. Another example of the unclear relationship between acid deposition and DOC trends is in the tallgrass prairie sites (KNZ) with positive DOC trends in streams affected by acid deposition. The site is in a karst landscape and well buffered against increases in hydrogen ion concentrations in soils and streams. Other directional changes at KNZ include increased woody vegetation in riparian zones (Veach et al., 2014) and increased drying in intermittent streams (Dodds et al., 2012) which could lead to changes in instream C concentrations (Rüegg et al., 2015).

A putative hypothesis about DOM properties is that concentrations of DOC and DON are highly correlated (e.g., Campbell et al., 2000; Goodale et al., 2000; Kortelainen et al., 2006; Lepistö et al., 2008; Mann et al., 2012). For those sites showing significant temporal trends, changes in concentrations of DON did not always track those of DOC, with decoupled trends found in over 50% of the analyses. The lack of temporal synchronicity in the changes of DOC and DON concentrations is likely the result of diverse sources of DOM captured among sites, the variable state factors (*sensu lato* Jenny, 1941) represented in this analysis, and variable biogeochemical processing along flow paths and stream networks (McDowell et al., 2004; Yates et al., 2019). Across the array of streams where DOM stoichiometry is significantly changing, DOM is becoming enriched with C and relatively depleted in N suggesting that fundamental changes in the energy and nutrient balance of freshwater ecosystems is occurring over large spatial scales.

### 4.1 Cross biome patterns in DOC and DON concentration trends

Many studies examining the response of DOC over time are reported from regions exposed to significant amounts of acidic deposition (Driscoll et al., 2003; Hall et al., 2021; Hruška et al., 2009; Monteith et al., 2007; Worrall et al., 2004). Whereas these studies have informed the notion that DOC concentrations are increasing in northern temperate streams (and we present complementary results), we also show that the directional change in concentrations of DOC and DON is highly variable and site‐ and stream‐specific. Our DOC Sen slopes are within the range of variability reported from other studies that report values ranging between −0.25 and 0.51 mg C/L per year in streams primarily from northern latitudes (Clair et al., 2008; Coble et al., 2018; De Wit et al., 2007; Driscoll et al., 2003; Evans et al., 2005). And while the DON Sen slopes presented here are within the range of those reported earlier (0.0027–0.003 mg N/L per year in northern latitudes [Clair et al., 2008; Lepistö et al., 2008]), we also present negative DON trends. Studies addressing the long‐term trends in DON are rarer than those of DOC, necessitating a broader assessment of DON trends. Our results suggest that changes in DOM composition may have the greatest impact in ecosystems with the lowest DOM concentrations such as tall grass prairies (KNZ) and tropical rainforest (LUQ). In these ecosystems with low DOM concentration, small changes in DOC and DON can create a large proportional change with potentially meaningful ramifications for stream metabolic regimes (Bernhardt et al., 2018) and biogeochemical reaction rates that are often limited by the availability of energy (Brailsford, Glanville, Golyshin, Johnes, et al., 2019; Brailsford, Glanville, Golyshin, Marshall, et al., 2019; Rodríguez‐Cardona et al., 2021).

**FIGURE 5:**
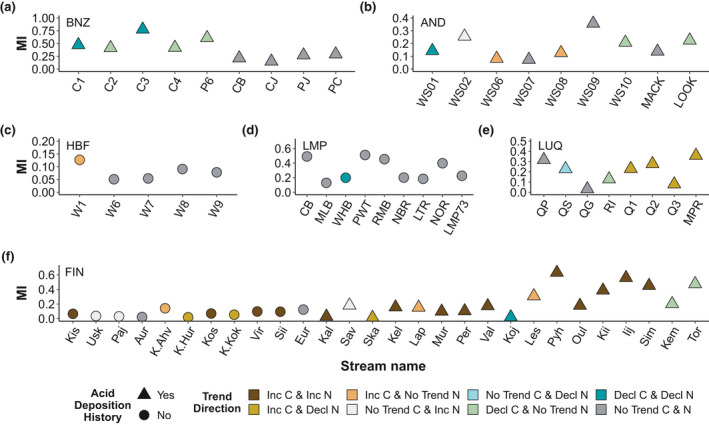
Mutual information values (MI) for DOC and DON. Shapes describe the acid deposition history by site, circles represent sites with a history of acid deposition and triangles are for sites that have not been affected by acid deposition. Colors represent the directional trend of DOC and DON based on their Sen slope where no trend are streams with a Sen slope with *p* > .05. Note Konza and 4 Finnish streams (YLIJOKI 1, Vuoksi Vastuupuomi 061, KOTIOJA 1, and Kivipuro 39) were excluded due to limited DON data available for these streams

Contrary to our hypothesis, synchronous changes in concentrations of DOC and DON were only found in a small number of streams (Figure 5). Asynchronous changes in concentrations of DOC and DON suggest different controls on the C‐rich and N‐rich fractions of the DOM pool and/or different drivers of DOC and DON flux to streams. We found numerous examples where concentrations of DOC and DON changed in opposite directions, demonstrating that the DOM pool as a whole is highly dynamic and that the different constituents of DOM do not always have the same ecological and biogeochemical sources and roles (e.g., Bernal et al., 2005; Brookshire et al., 2007; Lutz et al., 2011; McDowell et al., 2004; Wymore et al., 2015, 2018; Yates et al., 2019). For example, we found streams increasing in DOC but decreasing in DON (Figure 5) as well as sites that changed in either DOC or DON, but not in the other constituent. These scenarios suggest a biogeochemical decoupling of the C‐rich and N‐rich fractions of the DOM pool where DON cycling has little effect on the overall DOC pool. Changes in concentrations of DON with no significant trend in concentrations of DOC may be the result of DON being more mobile and reactive along flow paths relative to DOC due to its hydrophilic nature (Aiken et al., 1992; Hood et al., 2003; Inamdar et al., 2012;). Scenarios in which no significant trend in DOC concentrations occurs but DON concentrations decline could also occur in the nutrient limited systems where both terrestrial and aquatic biota mine the N contained within DOM (Brailsford, Glanville, Golyshin, Marshall, et al., 2019; Jones et al., 2005; Kissman et al., 2017; Mackay et al., 2020; Neff et al., 2003; Wymore et al., 2015). We did not detect any instances where DOC concentration is declining but DON concentration is increasing, suggesting that autochthonous contributions to the DON pool are small compared to heterogeneous terrestrial inputs from the watershed, at least for the streams included in this study.

### 4.2 A changing stoichiometry of the DOM pool

DOC and DON concentrations represent two ways to measure the composition of the DOM pool, yet few studies have used both bulk elemental analyses as a way to describe the heterogenous DOM pool (McDowell et al., 2019). Although a stoichiometric approach to understanding nutrient and elemental cycling has a rich history (Elser et al., 2000; Redfield, 1958), the principles have seldom been applied to understanding changes in bulk DOM composition over time. For those sites where a significant change in DOC:DON stoichiometry was detected, the predominant direction of change reflected the C‐enrichment or N‐depletion of DOM. The exception to this general pattern was in streams at the LMP site, located in the temperate deciduous forests of New England (Wymore et al., 2021a), where DOC:DON ratios are decreasing, indicating the relative N‐enrichment of DOM. These sites have a high percentage of wetlands (Flint & McDowell, 2015), which are likely contributing to these changing stoichiometric ratios (Coble et al., 2019). N‐enriched DOM may provide additional nutrients to microbial communities making more NH_4_^+^ available through mineralization. In turn, competition for dissolved inorganic N may decline with higher rates of nitrification and increased NO_3_^−^ production and export (Wymore et al., 2019), while increasing DON concentrations instream may provide an alternative nutrient resource for uptake by the primary producers (Mackay et al., 2020). In contrast, streams with increasing DOC:DON ratios may reflect increasing watershed N demand from greater retention in soils and increasing vegetative growth, possibly from CO_2_ enrichment (Craine et al., 2018; Groffman et al., 2018; Huang et al., 2015). Just as instream primary producers can take up DON compounds directly as a nutrient resource, trees can bypass microbial symbionts taking up labile forms of DON directly from soils (Neff et al., 2003), which in turn would decrease DON exports to streams leading to increases in DOC:DON ratios. Changes in DOC:DON ratios can alter rates of N transformations including nitrification (Strauss & Lamberti, 2002), and NO_3_^−^ concentrations (Bernhardt & McDowell, 2008). While the ecosystem and biogeochemical consequences of changes in DOC:DON is a relatively understudied topic, stoichiometric shifts in this particular compartment of organic matter will likely influence other biogeochemical cycles (Wymore et al., 2019; Yates et al., 2019), driving changes in the aquatic ecosystem and downstream, creating nutrient export regimes that can affect trophic assemblages in receiving bodies of water (Schade et al., 2005).

### 4.3 Atmospheric deposition

**FIGURE 6:**
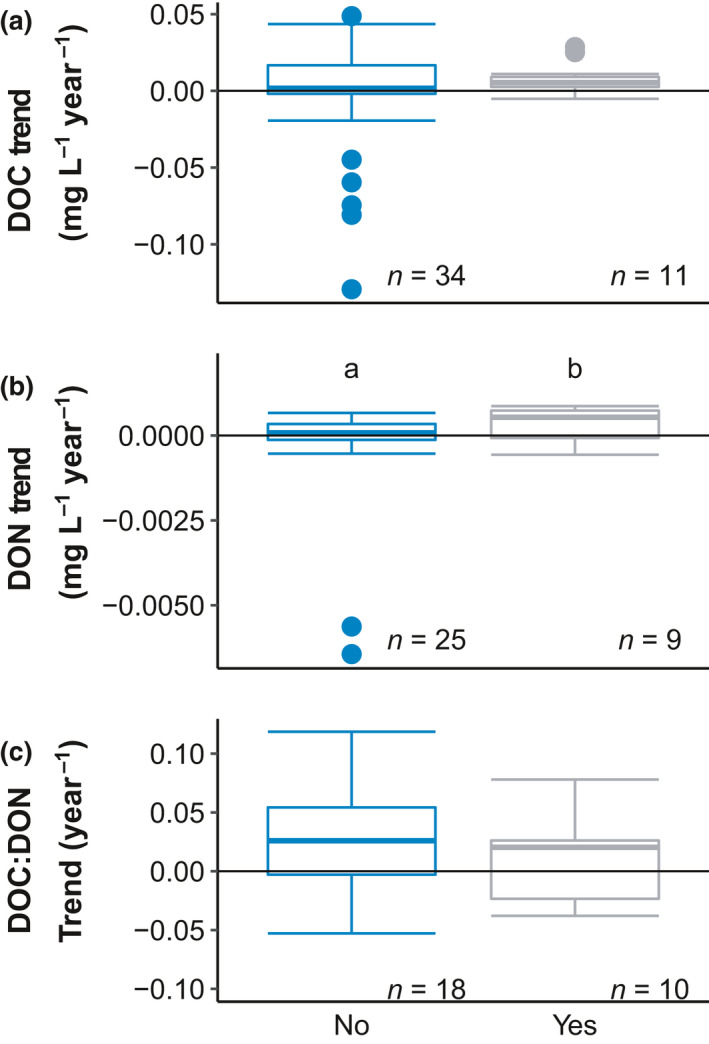
Significant Sen slopes (*p* < .05) in (a) DOC, (b) DON, and (c) DOC:DON ratios grouped by history of acid deposition where sites affected by acid deposition are in grey (FIN, HBF, LMP, KNZ) and sites not affected by acid deposition (CPC, AND, LUQ) are in blue. There is an outlier point in the DOC:DON trends for sites not affected by acid deposition that was excluded from the figure and statistics, the value is 0.96 year^−1^ from HBF. Letters denote statistically significant differences determined by Kruskall–Wallis rank‐sum test (DOC *p* = .38; DON *p* = .05, and DOC:DON *p* = .56). *p*‐values for one‐sample *t*‐test, to determine if means are different from 0 for DOC in acid deposition affected sites *p* = .008 and no acid deposition *p* = .35; DON in acid deposition affected sites *p* = .05 and no acid deposition *p* = .41; DOC:DON in acid deposition affected sites *p* = .29 and no acid deposition *p* = .008

In sites historically affected by acid deposition such as HBF, LMP, and Southern Finland, Sen slopes are mostly increasing for DOC and DON concentrations, consistent with previous studies in Northeast regions of the United States, Europe, and United Kingdom (Couture et al., 2012; Driscoll et al., 2003; Evans et al., 2005; Gavin et al., 2018; Monteith et al., 2007; Worrall et al., 2004). Similar trends for DOC and DON in these sites suggest analogous watershed sources, flow paths, and release mechanisms for both the C‐rich and N‐rich fractions of DOM. In these acid‐impacted sites, the rates of change for DOC and DON concentrations remain proportional, leading to a stoichiometrically stable DOM pool, despite the large changes in DOC concentrations (Wymore et al., 2021c). Sites affected by atmospheric acid deposition generally show increases in DON evidenced by the significant one‐sample *t* tests (Figure 6).

Conversely, sites not affected by acid deposition generally show declines in DON, falling below the DON zero line (Figure 6). These results highlight the importance of DON as a component of the DOM pool, and its sensitivity to changes in N loading from the watershed. Sites unaffected by acid deposition exhibit the greatest change in DOC:DON ratios, very likely driven by larger declines in DON concentration relative to DOC, as demonstrated by the decoupling of DOC and DON (Figure 5). Recovery from atmospheric deposition plays an important role in the delivery of DOM to aquatic ecosystems but appears to have little effect on the stoichiometry of DOM.

### 4.4 Predictors of DOM trends

The ambient stream chemistry models for all DOM trend models selected major dissolved ions such as Ca^2+^. This result supports the idea that these streams are recovering from acid deposition and as soil Ca^2+^ recovers, DOM declines due to decreased DOM solubility (Miller et al., 2016). For both DOC and DON models, ambient mean DOC and DON concentrations, respectively, were selected suggesting that streams with greater DOC or DON concentrations will experience the greatest changes over time. Ambient NO_3_^−^ concentrations had the second‐highest beta coefficients for DON trends model, demonstrating the connection between the organic and inorganic N pool in streams and how DIN can influence concentrations of DON (Wymore et al., 2015). Either peak NO_3_^−^ or SO_4_^−^ deposition were selected for all DOM trends in the atmospheric acid deposition models demonstrating the lasting effects and recovery of acid deposition on aquatic ecosystems, but for DOC:DON trends this model explained very little of the variance. This result suggests that although atmospheric acid deposition can influence DOC and DON concentrations, the proportional changes might not be large enough to be detected in stoichiometry, at least for the subset of streams selected in this model.

In the watershed characteristics models, mean annual temperature (MAT) and watershed elevation were selected for DOC and DOC:DON trends demonstrating that the geographical location of the streams (MAT as a surrogate for latitudinal changes), can have a strong influence on DOM over time. DOC and DON trends were greatest in granitic watersheds as this bedrock has a poor buffering capacity (Robinson, 1997) allowing a greater release of DOM to adjacent aquatic systems. This translates to an opposite pattern for DOC:DON ratios where they were lower in granitic watersheds and greater in the volcaniclastic watersheds (Figure S4). The type of soil also played a role in DOM trends being streams in silty and sandy loam landscapes the ones showing the lowest DOC and DON trends (Figure S5). This finding suggests that the adsorption to silt particles can influence DOM availability by controlling the long‐term storage and export of DOC and DON (Dosskey & Bertsch, 1997; Kaiser & Guggenberger, 2000). The higher trends of DOC and DON in moraine sites (Figure S5) could be due to greater OM availability and associated microbial decomposition activity (Bruhn et al., 2021). Collectively, the results of these models support the hypothesis that regional state factors such as geology and soil type are important controls of stream long‐term DOM trends.

## 5 CONCLUSION

This study adds to the growing body of literature on long‐term trends of stream water DOM by expanding the scope of past studies through the inclusion of underrepresented biomes and ecosystems including tropical rainforests, arctic taiga, and tallgrass prairie. Our study also provides one of the first large‐scale assessments of long‐term trends in concentrations of DON and DOM stoichiometry in streams. We demonstrated increases in DOC concentrations in sites recovering from acid deposition, similar to previous findings, and we have shown that those are accompanied by proportional increases in DON. In addition, there can be increasing trends in DOC in sites with no atmospheric acid deposition history where in these cases, trends are associated with local state factors such as soil and geology. Although we have shown that positive trends in DOC can occur regardless of the acid deposition history, we also demonstrate that increases in DOC are not ubiquitous across broad latitudinal gradients. Declining trends in DON suggest on the differential cycling of DOC and DON in soils and within streams. The biomes in which streams are embedded are expected to influence the biogeochemistry of those systems (Dodds et al., 2019), but exactly how is poorly known. Changes in DOC and DON concentrations will have implications for in‐stream biogeochemical processes as well as the bulk composition of DOM exports to receiving bodies of water, especially those systems where changes in DOC and DON are decoupled. Continued monitoring of these long‐term trends in DOM concentration and stoichiometry in response to climatic and landscape attributes is important to better understand the ultimate fate of DOM and nutrients in freshwater ecosystems in the face of global change.

## AUTHOR CONTRIBUTIONS

Bianca M. Rodríguez‐Cardona and Adam S. Wymore conceived the project. Bianca M. Rodríguez‐Cardona, Adam S. Wymore, Ashley A. Coble, and Carla López‐Lloreda, organized and synthesized the data, and Jeremy B. Jones, Sherri L. Johnson, Pirkko Kortelainen, Walter K. Dodds, and William H. McDowell provided data. Bianca M. Rodríguez‐Cardona, Adam S. Wymore, Alba Argerich analyzed the data. All authors helped to interpret that data. Bianca M. Rodríguez‐Cardona wrote the initial draft of the manuscript with significant input from Adam S. Wymore, Robert G. M. Spencer, Ashley M. Helton, Susana Bernal, and William H. McDowell. All co‐authors discussed hypotheses, data and results, and commented and revised the manuscript.

## Supporting information

Supplementary Material

## Data Availability

The data used in these analyses represent a synthesis of multiple data sets. The individual data sets and their associated repositories and references can be found in Tables S4 and S5. The archived data set (Wymore et al., 2021b) is openly shared at the Environmental Data Initiative (EDI) according to FAIR principles of data sharing (https://environmentaldatainitiative.org). Data can be accessed here: https://doi.org/10.6073/pasta/50965f9e091ffa833da3c73bce2467fa.
