# Supplementary material for: Shifting stoichiometry: Long‐term trends in stream‐dissolved organic matter reveal altered C:N ratios due to history of atmospheric acid deposition
Source: Glob Chang Biol. 2021 Nov 5;28(1):98–114. doi: 10.1111/gcb.15965 (PMC9297910; doi:10.1111/gcb.15965)
Supplement: Supplementary file 1 — Supplementary Material [file GCB-28-98-s001.docx]

**Supporting Information**

Shifting Stoichiometry: Long-term trends in stream dissolved organic matter reveal altered C:N ratios due to history of atmospheric acid deposition

Bianca M. Rodríguez-Cardona^1,16*^, Adam S. Wymore^1^, Alba Argerich^2^, Rebecca T. Barnes^3^, Susana Bernal^4^, E. N. Jack Brookshire^5^, Ashley A. Coble^6^, Walter K. Dodds^7^, Hannah M. Fazekas^1^, Ashley M. Helton^8^, Penny J. Johnes^9^, Sherri L. Johnson^10^, Jeremy B. Jones^11^, Sujay S. Kaushal^12^, Pirkko Kortelainen^13^, Carla López-Lloreda^1,14^, Robert G. M. Spencer^15^, & William H. McDowell

**
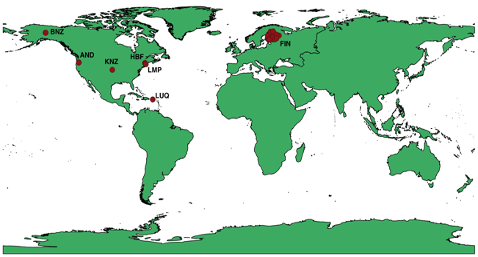
**

**Figure S1** Location of all sites across the Northern Hemisphere, see Table 1 for complete site names.

**Figure S2**. Relationship between trends of (a) DOC, (b), DON, (c) DOC:DON and the total record length of years used to calculate DOM trends. Each point is an individual stream for each site. R^2^ and p -values were determined from simple linear regressions.

**Figure S3.** Time series of monthly SO_4_ (Yellow) and NO_3_ (blue) atmospheric deposition from National Atmospheric Deposition Program collection sites at (**a**) Caribou-Poker Creeks Research Watershed, (**b**) Hubbard Brook Experimental Forest, (**c**) H.J. Andrews Experimental Forest, (**d**) Konza Prairie, (**e**) and Luquillo Experimental Forest. The grey box corresponds to the length of records for stream water DOC and DON concentrations and DOC:DON molar ratios analyzed for trends in this study. Note different y-axes.

Fig S4. Boxplots of DOC (p=0.22), DON (0.01), DOC:DON (p=0.005) trends (significant Sen slopes, p<0.05) by watershed geology. Geology types with n=1 were excluded from Kruskall-Wallis test. An outlier for DOC:DON trends near 1 yr^-1^ was removed, but differences were still significant when it was included (p=0.01).

Fig S5. Boxplots of DOC (p=0.0001), DON (p=0.002), DOC:DON (p=0.06) trends (significant Sen slopes, p<0.05) by watershed soil type. Soil types with n=1 were excluded from Kruskall-Wallis test.

Fig S6. Boxplots of (a) DOC (p=0.00001), DON (p=0.0001), DOC:DON (p=0.03) trends (significant Sen slopes, p<0.05) by watershed forest type. Forest types with n=1 were excluded from Kruskall-Wallis test.

**Table S1**. Individual stream (with abbreviated stream name) characteristics such as latitude (Lat) and longitude (Long), mean annual temperature (MAT), mean annual precipitation (MAP), watershed elevation (Elev) and area along with mean stream pH, temperature (Temp), and Conductivity (Cond). Reference describe sites that are specifically labeled reference watersheds, or no management or manipulations have been documented for these watersheds. Forested in Finland sites are less impacted than those described as suburban. – denote data is not available for that stream.

| **Site** | **River Name** | **Stream Name** | **Abbrev. Stream Name** | **Lat.** | **Long.** | **MAT**  **(°C)** | **MAP**  **(mm)** | **Elev.**  **(m)** | **Area**  **(km^2^)** | **pH** | **Temp**  **(°C)** | **Cond**  **(µs/cm)** | **Land-use History** | **Anthropogenic**  **Activity** |
| --- | --- | --- | --- | --- | --- | --- | --- | --- | --- | --- | --- | --- | --- | --- |
| FIN | Aurajoki | Aura 54 ohikulku va6401 | Aur | 60.47 | 22.36 | 5.1 | 660 | 10 | 874 | - | - | - | Suburban | Disturbed |
| FIN | Eurajoki | Eura 42 Pori-Rma va6900 | Eur | 61.20 | 21.73 | 4.3 | 622 | 9 | 1336 | - | - | - | Suburban | Disturbed |
| FIN | Iijoki | Iijoki Raasakan voimal | Iij | 65.33 | 25.42 | 1.6 | 474 | 17 | 14191 | - | - | - | Forested | Forested |
| FIN | Kalajoki | Kalajoki 11000 | Kal | 61.95 | 27.22 | 2.3 | 524 | 110 | 13 | - | - | - | Agriculture | Disturbed |
| FIN | - | Kelopuro 28 | Kel | 63.16 | 30.69 | 3.2 | 554 | 166 | 0.76 | - | - | - | Forested | Forested |
| FIN | Kemijoki | KEMIJOKI ISOHAARA 14000 | Kem | 65.79 | 24.55 | 1.3 | 516 | 13 | 51127 | - | - | - | Forested | Forested |
| FIN | Kiiminginjoki | Kiiminkij 13010 4-tien s | Kii | 65.18 | 25.36 | 1.7 | 468 | 0 | 3814 | - | - | - | Forested | Forested |
| FIN | Kiskonjoki | Kisko 14 Vanhak va6111 | Kis | 60.13 | 23.16 | 5.1 | 612 | 12 | 1047 | - | - | - | Agriculture | Disturbed |
| FIN | - | Kivipuro 39 | Kiv | 63.87 | 28.65 | 3.2 | 554 | 186 | 0.54 | - | - | - | Forested | Forested |
| FIN | Kokemäenjoki | Kojo 35 Pori-Tre | Koj | 63.88 | 28.67 | 4.2 | 615 | 210 | 1.20 | - | - | - | Agriculture | Forested |
| FIN | Koskenkylänjoki | Koskenkylänjoki 3,0 6030 | Kos | 60.50 | 25.95 | 3.2 | 554 | 9 | 895.00 | - | - | - | Agriculture | Disturbed |
| FIN | - | Kotioja 1 | Kot | 66.14 | 26.15 | 3.2 | 554 | 162 | 18.00 | - | - | - | Forested | Forested |
| FIN | Kymijoki | Kymij Huruksela 033 5600 | K.Hur | 60.49 | 26.45 | 4.7 | 608 | 20 | 37159 | - | - | - | Forested | Forested |
| FIN | Kymijoki | Kymijoki Ahvenkoski 001 | K.Ahv | 60.49 | 26.45 | 3.2 | 554 | 0 | - | - | - | - | Forested | Forested |
| FIN | Kymijoki | Kymijoki Kokonkoski 014 | K.Kok | 60.53 | 26.91 | 3.2 | 554 | 12 | - | - | - | - | Suburban | Disturbed |
| FIN | Lapuanjoki | Lapuanjoki 9900 | Lap | 63.53 | 22.53 | 2.9 | 536 | 9 | 4122 | - | - | - | Agriculture | Disturbed |
| FIN | Lestijoki | Lestijoki 10800 8-tien s | Les | 64.06 | 23.66 | 2.5 | 528 | 5 | 1373 | - | - | - | Suburban | Disturbed |
| FIN | - | Murtopuro 42 | Mur | 63.76 | 28.49 | 3.2 | 554 | 179 | 4.90 | - | - | - | Forested | Forested |
| FIN | Oulujoki | Oulujoki 13000 | Oul | 65.02 | 25.47 | 1.9 | 460 | 0 | 22845 | - | - | - | Forested | Forested |
| FIN | Paimionjoki | Pajo 44 Isosilta va6301 | Paj | 60.46 | 22.68 | 4.9 | 640 | 10 | 1088 | - | - | - | Agriculture | Forested |
| FIN | Perhonjoki | Perhonjoki 10600 | Per | 63.85 | 23.22 | 2.6 | 534 | 5 | 2524 | - | - | - | Suburban | Disturbed |
| FIN | Pyhäjoki | Pyhäjoki Hourunk 11400 | Pyh | 64.46 | 24.27 | 3.2 | 554 | 10 | 3712 | - | - | - | Suburban | Disturbed |
| FIN | - | Savi 12 mittapato | Sav | 62.51 | 26.03 | 3.2 | 554 | 94 | 5.39 | - | - | - | Agriculture | Disturbed |
| FIN | Siikajoki | Siikajoki 8-tien s 11600 | Sii | 60.60 | 22.67 | 2.1 | 486 | 51 | 15.40 | - | - | - | Forested | Forested |
| FIN | Simojoki | Simojoki as. 13500 | Sim | 65.66 | 25.08 | 1.5 | 490 | 9 | 4218 | - | - | - | Forested | Forested |
| FIN | Kyrönjoki | Skatila vp 9600 | Ska | 63.09 | 21.89 | 3.4 | 525 | 3 | 3160 | - | - | - | Forested | Forested |
| FIN | Tornionjoki | Tornionj kukkola 14310 | Tor | 65.96 | 24.05 | 1.1 | 540 | 21 | 4923 | - | - | - | Forested | Forested |
| FIN | Uskelanjoki | Uske 16 Salon yp va6101 | Usk | 60.39 | 23.13 | 4.8 | 618 | 3 | 566 | - | - | - | Agriculture | Disturbed |
| FIN | - | Valipuro 38 | Val | 63.87 | 28.66 | 3.2 | 554 | 186 | 0.86 | - | - | - | Forested | Forested |
| FIN | Virojoki | Virojoki 006 3020 | Vir | 60.58 | 27.71 | 4.6 | 624 | 9 | 357 | - | - | - | Agriculture | Disturbed |
| FIN | - | Vuoksi Vastuupuomi 061 | Vuo | 61.20 | 28.78 | 3.8 | 671 | 70 | 61466 | - | - | - | Forested | Forested |
| FIN | - | Ylijoki 1 | Ylij | 66.15 | 26.16 | 3.2 | 554 | 164 | 56 | - | - | - | Forested | Forested |
| CPC | - | C1 | C1 | 65.15 | -147.65 | -4.3 | 312 | 537 | 6.70 | 7.42 | 4.00 | 53.3 | Reference | Forested |
| CPC | - | C2 | C2 | 65.16 | -147.60 | -4.3 | 312 | 375 | 5.20 | 7.66 | 4.90 | 78.7 | Reference | Forested |
| CPC | - | C3 | C3 | 65.14 | -147.57 | -4.3 | 312 | 357 | 5.70 | 7.54 | 3.80 | 72.7 | Reference | Forested |
| CPC | - | C4 | C4 | 65.16 | -147.50 | -4.7 | 309 | 267 | 10.00 | 7.82 | 5.09 | 103 | Burned 1999 | Forested |
| CPC | - | CB | CB | 65.10 | -147.40 | -5.4 | 296 | 250 | 16.70 | 7.55 | 5.00 | 67.4 | Reference | Forested |
| CPC | - | CJ | CJ | 65.15 | -147.49 | -4.7 | 309 | 224 | 41.70 | 7.60 | 6.50 | 82 | Reference | Forested |
| CPC | - | P6 | P6 | 65.18 | -147.39 | -5.6 | 294 | 465 | 7 | 7.82 | 4.18 | 123 | Burned 1999 | Forested |
| CPC | - | PC | PC | 65.15 | -147.48 | -4.7 | 309 | 217 | 101.50 | 7.81 | 8.00 | 102.5 | Burned 1999 | Forested |
| CPC | - | PJ | PJ | 65.15 | -147.48 | -4.7 | 309 | 222 | 59.80 | 7.85 | 8.49 | 111 | Reference | Forested |
| HBF | - | Watershed 1 | W1 | 43.95 | -71.73 | 4.8 | 1160 | 482 | 0.12 | 5.53 | 8.00 | 13 | Wallastonite Additions | Disturbed |
| HBF | - | Watershed 6 | W6 | 43.95 | -71.74 | 4.8 | 1160 | 546 | 0.13 | 5.21 | 5.50 | 12.5 | Reference | Forested |
| HBF | - | Watershed 7 | W7 | 43.93 | -71.77 | 4.8 | 1160 | 614 | 0.77 | 5.99 | 4.80 | 12.6 | Reference | Forested |
| HBF | - | Watershed 8 | W8 | 43.93 | -71.76 | 4.8 | 1160 | 602 | 0.59 | 5.66 | 4.60 | 12.9 | Reference | Forested |
| HBF | - | Watershed 9 | W9 | 43.93 | -71.75 | 4.8 | 1160 | 696 | 0.68 | 4.64 | 3.40 | 17.9 | Reference | Forested |
| LMP | - | College Brook | CB | 43.14 | -70.94 | 8.9 | 1169 | 17 | 2.27 | 7.16 | 10.74 | 524 | Urban | Disturbed |
| LMP | - | Lamprey River | LMP73 | 43.10 | -70.95 | 8.9 | 1169 | 14 | 479.20 | 6.70 | 11.93 | 87 | Suburban | Forested |
| LMP | - | Little River | LTR | 43.11 | -71.01 | 8.9 | 1169 | 26 | 51.70 | 6.69 | 9.55 | 62.9 | Suburban | Forested |
| LMP | - | Moonlight Brook | MLB | 43.08 | -70.94 | 8.9 | 1169 | 4 | 0.90 | 6.58 | 9.48 | 455 | Urban | Disturbed |
| LMP | - | North Branch | NBR | 43.06 | -71.24 | 8.9 | 1169 | 64 | 41.50 | 6.46 | 10.86 | 74 | Suburban | Forested |
| LMP | - | North River | NOR | 43.08 | -71.04 | 8.9 | 1169 | 30 | 128.90 | 6.62 | 11.21 | 61 | Suburban | Forested |
| LMP | - | Pawtuckaway | PWT | 43.10 | -71.20 | 8.9 | 1169 | 136 | 2.60 | 5.88 | 12.16 | 25 | Forest | Forested |
| LMP | - | Rum Brook | RMB | 43.05 | -71.03 | 8.9 | 1169 | 30 | 4.90 | 6.68 | 9.84 | 113 | Suburban | Forested |
| LMP | - | Wednesday Hill Brook | WHB | 43.12 | -71.00 | 8.9 | 1169 | 32 | 1.00 | 7.09 | 9.46 | 221 | Suburban | Disturbed |
| AND | - | Lookout Creek | LOOK | 44.28 | -122.10 | 7.5 | 1950 | 422 | 62.42 | 7.40 | - | 31.3 | Partially Harvested | Forested |
| AND | - | Mack Creek | MACK | 44.22 | -122.17 | 7.5 | 1950 | 755 | 5.81 | 7.30 | - | 26.75 | Partially Harvested | Forested |
| AND | - | Watershed 1 | WS01 | 44.21 | -122.26 | 7.5 | 1950 | 439 | 0.96 | 7.50 | - | 43.1 | Clear-cut | Forested |
| AND | - | Watershed 2 | WS02 | 44.21 | -122.23 | 7.5 | 1950 | 545 | 0.60 | 7.50 | - | 36.45 | Reference | Forested |
| AND | - | Watershed 6 | WS06 | 44.27 | -122.18 | 7.5 | 1950 | 878 | 0.13 | 7.50 | - | 38.6 | Clear-cut | Forested |
| AND | - | Watershed 7 | WS07 | 44.27 | -122.17 | 7.5 | 1950 | 918 | 0.15 | 7.50 | - | 42.9 | Partial Overstory Harvest | Forested |
| AND | - | Watershed 8 | WS08 | 44.27 | -122.17 | 7.5 | 1950 | 962 | 0.21 | 7.50 | - | 38.3 | Reference | Forested |
| AND | - | Watershed 9 | WS09 | 44.20 | -122.25 | 7.5 | 1950 | 426 | 0.09 | 7.50 | - | 48.45 | Reference | Forested |
| AND | - | Watershed 10 | WS10 | 44.22 | -122.25 | 7.5 | 1950 | 461 | 0.10 | 7.50 | - | 43.6 | Clear-cut | Forested |
| KNZ | - | Elder Spring | edlr | 39.10 | -96.61 | 12.1 | 866 | 337 | - | - | - | - | Reference | Forested |
| KNZ | - | Hiking Trail | hikx | 39.11 | -96.61 | 12.1 | 866 | 324 | - | - | - | - | Agriculture | Forested |
| LUQ | - | Bisley 1 | Q1 | 18.32 | -65.75 | 22.8 | 1768 | 377 | 0.07 | 7.26 | 23.00 | 97.4 | Reference | Forested |
| LUQ | - | Bisley 2 | Q2 | 18.31 | -65.75 | 22.8 | 1768 | 218 | 0.06 | 7.29 | 22.90 | 92.6 | Reference | Forested |
| LUQ | - | Bisley 3 | Q3 | 18.31 | -65.75 | 22.8 | 1768 | 198 | 0.35 | 7.32 | 22.63 | 85.6 | Reference | Forested |
| LUQ | - | Qda. Guaba | QG | 18.28 | -65.79 | 21.4 | 3229 | 642 | 0.13 | 6.75 | 20.80 | 47 | Reference | Forested |
| LUQ | - | Qda. Prieta | QP | 18.32 | -65.82 | 22 | 4587 | 431 | 0.31 | 7.02 | 22.00 | 79.6 | Reference | Forested |
| LUQ | - | Qda. Sonadora | QS | 18.32 | -65.82 | 22 | 3495 | 740 | 2.60 | 6.90 | 21.60 | 49.8 | Reference | Forested |
| LUQ | - | Río Icacos | RI | 18.28 | -65.79 | 21.4 | 2254 | 686 | 3.30 | 6.76 | 21.00 | 55.5 | Reference | Forested |
| LUQ | - | Mameyes Puente Roto | MPR | 18.33 | -65.75 | 23 | 2843 | 498 | 17.7 | 7.44 | 23.3 | 94.5 | Reference | Forested |

**Table S2**. Mean ambient solute concentrations for every stream. – denote data is not available for that stream.

| **Site** | **Stream** | **DOC**  **(mg/L)** | **DON**  **(mg/L)** | **TDN**  **(mg/L)** | **NO_3_^-^**  **(mg/L)** | **NH_4_^+^**  **(mg/L)** | **PO_4_^3-^**  **(mg/L)** | **Si**  **(mg/L)** | **TDP**  **(mg/L)** | **Na^+^**  **(mg/L)** | **K^+^**  **(mg/L)** | **Ca^2+^**  **(mg/L)** | **Mg^2+^**  **(mg/L)** | **SO_4_^2-^**  **(mg/L)** | **Cl^-^**  **(mg/L)** |
| --- | --- | --- | --- | --- | --- | --- | --- | --- | --- | --- | --- | --- | --- | --- | --- |
| FIN | Aur | 13.30 | 0.64 | 2.09 | 1.40 | 58 | 81 | - | 0.04 | 8.00 | 3.20 | 10.80 | 6.90 | - | - |
| FIN | Eur | 9.31 | 0.51 | 1.52 | 0.84 | 210 | 17 | - | 0.01 | 7.40 | 3.20 | 13.80 | 5.60 | - | - |
| FIN | Iij | 9.69 | 0.32 | 0.37 | 0.04 | 8 | 8 | - | 0.01 | 1.70 | 0.60 | 2.90 | 1.00 | - | - |
| FIN | Kal | 19.95 | 0.69 | 1.33 | 0.60 | 85 | 40 | - | 0.04 | 4.60 | 2.30 | 6.70 | 4.00 | - | - |
| FIN | Kel | 9.50 | 0.22 | 0.25 | 0.01 | 9 | 2 | - | 0.00 | 1.00 | 0.30 | 1.10 | 0.20 | - | - |
| FIN | Kem | 7.60 | 0.28 | 0.33 | 0.04 | 12 | 6 | - | 0.01 | 1.70 | 0.60 | 4.40 | 1.30 | - | - |
| FIN | Kii | 14.06 | 0.42 | 0.51 | 0.06 | 8.50 | 11 | - | 0.02 | 2.20 | 0.70 | 3.30 | 1.30 | - | - |
| FIN | Kis | 9.50 | 0.53 | 0.95 | 0.44 | 25 | 21 | - | 0.02 | 3.80 | 1.60 | 6.70 | 2.80 | - | - |
| FIN | Kiv | 27.08 | 0.47 | 0.48 | 0.01 | 6 | 5 | - | 0.01 | 1.50 | 0.60 | 1.30 | 0.80 | - | - |
| FIN | Koj | 9.50 | 0.44 | 1.05 | 0.53 | 64 | 16 | - | 0.01 | 6.80 | 2.00 | 7.70 | 2.70 | - | - |
| FIN | Kos | 9.41 | 0.50 | 1.43 | 0.96 | 43 | 42 | - | 0.02 | 6.30 | 3.20 | 9.70 | 5.10 | - | - |
| FIN | Kot | 15.20 | 0.44 | 0.52 | 0.07 | 10 | 10 | - | - | 1.20 | 0.50 | 5.40 | 2.00 | - | - |
| FIN | K.Hur | 7.41 | 0.31 | 0.54 | 0.22 | 22 | 3 | - | 0.01 | 6.23 | 1.50 | 5.30 | 1.50 | - | - |
| FIN | K.Ahv | 7.32 | 0.33 | 0.57 | 0.24 | 22 | 4 | - | 0.01 | 6.20 | 1.60 | 5.50 | 1.60 | - | - |
| FIN | K.Kok | 7.13 | 0.31 | 0.55 | 0.23 | 21 | 3 | - | 0.01 | 6.40 | 1.60 | 5.40 | 1.50 | - | - |
| FIN | Lap | 19.00 | 0.63 | 1.53 | 0.70 | 230 | 34 | - | 0.02 | 6.50 | 3.10 | 8.50 | 4.00 | - | - |
| FIN | Les | 19.00 | 0.58 | 0.88 | 0.27 | 54 | 30 | - | 0.03 | 2.70 | 1.40 | 3.90 | 1.70 | - | - |
| FIN | Mur | 23.75 | 0.45 | 0.51 | 0.01 | 7.50 | 11 | - | 0.01 | 1.50 | 0.60 | 1.70 | 0.80 | - | - |
| FIN | Oul | 8.93 | 0.29 | 0.36 | 0.05 | 14 | 6 | - | 0.01 | 1.70 | 0.70 | 2.90 | 1.00 | - | - |
| FIN | Paj | 11.40 | 0.63 | 2.14 | 1.40 | 56 | 98 | - | 0.04 | 7.90 | 4.30 | 9.90 | 7.10 | - | - |
| FIN | Per | 19.00 | 0.58 | 1.05 | 0.36 | 110 | 30 | - | 0.02 | 2.70 | 1.50 | 4.60 | 1.70 | - | - |
| FIN | Pyh | 17.10 | 0.56 | 0.89 | 0.30 | 44 | 23 | - | 0.03 | 4.60 | 1.90 | 9.90 | 2.70 | - | - |
| FIN | Sav | 10.45 | 0.55 | 1.73 | 1.10 | 80 | 65 | - | 0.03 | 6.60 | 2.80 | 9.22 | 6.70 | - | - |
| FIN | Sii | 18.05 | 0.54 | 0.82 | 0.22 | 55 | 36 | - | 0.03 | 3.10 | 1.40 | 4.45 | 2.05 | - | - |
| FIN | Sim | 11.40 | 0.37 | 0.45 | 0.04 | 13 | 5 | - | 0.01 | 1.50 | 0.60 | 3.30 | 1.40 | - | - |
| FIN | Ska | 19.00 | 0.64 | 1.90 | 0.98 | 190 | 43 | - | 0.02 | 7.10 | 3.10 | 9.30 | 4.84 | - | - |
| FIN | Tor | 5.42 | 0.21 | 0.27 | 0.01 | 7 | 5 | - | 0.01 | 1.40 | 0.70 | 3.80 | 1.00 | - | - |
| FIN | Usk | 10.45 | 0.58 | 1.90 | 1.20 | 79.50 | 73 | - | 0.03 | 10.00 | 3.80 | 12.00 | 8.00 | - | - |
| FIN | Val | 30.35 | 0.48 | 0.52 | 0.01 | 7 | 3.25 | - | 0.01 | 1.10 | 0.30 | 0.80 | 0.50 | - | - |
| FIN | Vir | 14.25 | 0.57 | 0.92 | 0.34 | 30 | 16 | - | 0.02 | 3.30 | 1.60 | 5.80 | 1.70 | - | - |
| FIN | Vuo | 6.75 | 0.22 | 0.42 | 0.15 | 8 | 2 | - | 0.00 | 5.20 | 1.30 | 5.10 | 1.30 | - | - |
| FIN | Ylij | 13.11 | 0.44 | 0.57 | 0.06 | 19 | 12 | - | - | 1.30 | 0.60 | 5.25 | 2.00 | - | - |
| CPC | C1 | 3.66 | 0.24 | 0.53 | 0.27 | 27.17 | - | 15.11 | - | 0.97 | 0.33 | 7.65 | 1.57 | 3.18 | 0.28 |
| CPC | C2 | 2.63 | 0.25 | 0.82 | 0.56 | 26.61 | - | 21.76 | - | 1.04 | 0.37 | 10.81 | 3.18 | 6.41 | 0.33 |
| CPC | C3 | 5.01 | 0.30 | 0.71 | 0.43 | 26.61 | - | 16.16 | - | 1.18 | 0.38 | 11.07 | 1.82 | 7.17 | 0.27 |
| CPC | C4 | 2.28 | 0.24 | 0.87 | 0.65 | 25.21 | - | 21.31 | - | 1.30 | 0.57 | 15.71 | 3.01 | 7.08 | 0.32 |
| CPC | CB | 3.28 | 0.23 | 0.54 | 0.33 | 16.81 | - | 20.80 | - | 1.14 | 0.41 | 10.18 | 2.32 | 4.89 | 0.34 |
| CPC | CJ | 5.06 | 0.31 | 0.62 | 0.32 | 16.11 | - | 22.68 | - | 1.27 | 0.52 | 13.71 | 2.51 | 6.36 | 0.28 |
| CPC | P6 | 4.36 | 0.27 | 0.72 | 0.46 | 24.16 | - | 20.93 | - | 1.32 | 0.51 | 18.80 | 3.17 | 15.26 | 0.38 |
| CPC | PC | 4.74 | 0.28 | 0.54 | 0.27 | 15.97 | - | 23.50 | - | 1.27 | 0.58 | 15.92 | 3.16 | 8.40 | 0.39 |
| CPC | PJ | 5.10 | 0.33 | 0.60 | 0.26 | 21.01 | - | 22.47 | - | 1.38 | 0.66 | 17.18 | 3.44 | 9.27 | 0.41 |
| HBF | W1 | 2.28 | 0.08 | 0.28 | 0.17 | 4 | 0.3 | 5.29 | - | 0.69 | 0.11 | 1.18 | 0.16 | 0.98 | 0.43 |
| HBF | W6 | 1.94 | 0.07 | 0.12 | 0.01 | 4 | 0.3 | 4.26 | - | 0.77 | 0.15 | 0.63 | 0.21 | 1.21 | 0.41 |
| HBF | W7 | 1.68 | 0.06 | 0.10 | 0.02 | 4 | 0.3 | 5.20 | - | 0.77 | 0.15 | 0.91 | 0.30 | 1.19 | 0.37 |
| HBF | W8 | 2.63 | 0.07 | 0.13 | 0.04 | 4 | 0.7 | 5.80 | - | 0.83 | 0.16 | 0.76 | 0.30 | 1.18 | 0.38 |
| HBF | W9 | 7.52 | 0.14 | 0.21 | 0.02 | 4 | 0.3 | 6.10 | - | 0.71 | 0.16 | 0.62 | 0.21 | 1.15 | 0.39 |
| LMP | CB | 4.16 | 0.23 | 0.83 | 0.60 | 38.87 | 16.51 | 10.31 | - | 80.79 | 5.01 | 23.84 | 6.56 | 4.55 | 140.46 |
| LMP | LMP73 | 5.25 | 0.21 | 0.37 | 0.12 | 0.02 | 0.00 | 5.18 | - | 14.54 | 1.15 | 6.37 | 1.32 | 1.87 | 24.25 |
| LMP | LTR | 5.45 | 0.17 | 0.30 | 0.10 | 0.01 | 0.00 | 7.17 | - | 11.93 | 0.79 | 5.04 | 1.16 | 1.38 | 20.16 |
| LMP | MLB | 1.64 | 0.09 | 0.79 | 0.70 | 0.04 | 0.00 | 10.85 | - | 70.66 | 3.86 | 37.53 | 6.56 | 4.47 | 163.60 |
| LMP | NBR | 5.03 | 0.18 | 0.25 | 0.05 | 0.01 | 0.00 | 6.39 | - | 14.36 | 0.73 | 4.98 | 1.05 | 1.62 | 24.55 |
| LMP | NOR | 5.59 | 0.20 | 0.28 | 0.05 | 0.01 | 0.00 | 6.42 | - | 10.53 | 0.95 | 5.44 | 1.03 | 1.82 | 16.68 |
| LMP | PWT | 5.48 | 0.19 | 0.24 | 0.01 | 0.01 | 0.00 | 7.87 | - | 2.06 | 0.76 | 3.98 | 1.01 | 1.00 | 2.49 |
| LMP | RMB | 5.56 | 0.21 | 0.38 | 0.11 | 0.02 | 0.01 | 10.67 | - | 20.59 | 2.14 | 10.80 | 1.67 | 2.16 | 36.84 |
| LMP | WHB | 3.07 | 0.14 | 0.81 | 0.65 | 0.01 | 0.00 | 9.05 | - | 32.23 | 1.39 | 16.18 | 6.70 | 3.59 | 50.46 |
| AND | LOOK | 0.90 | 0.03 | 0.04 | 0.00 | 6.00 | 11.00 | 7.78 | 0.01 | 2.07 | 0.40 | 3.00 | 0.88 | 0.08 | 0.67 |
| AND | MACK | 0.84 | 0.03 | 0.09 | 0.05 | 8.00 | 7.00 | 6.80 | 0.02 | 1.49 | 0.34 | 2.46 | 0.87 | 0.10 | 0.65 |
| AND | WS01 | 1.14 | 0.05 | 0.08 | 0.01 | 13 | 21.00 | 9.10 | 0.03 | 2.64 | 0.20 | 4.64 | 1.02 | 0.18 | 0.74 |
| AND | WS02 | 1.47 | 0.03 | 0.03 | 0.00 | 6 | 22.00 | 9.00 | 0.04 | 2.54 | 0.38 | 3.47 | 0.82 | 0.12 | 0.90 |
| AND | WS06 | 0.52 | 0.02 | 0.03 | 0.00 | 5 | 13.00 | 8.20 | 0.03 | 2.11 | 0.38 | 3.94 | 0.99 | 0.07 | 0.61 |
| AND | WS07 | 0.53 | 0.02 | 0.02 | 0.00 | 4 | 22.00 | 9.32 | 0.03 | 2.32 | 0.58 | 4.07 | 1.12 | 0.05 | 0.60 |
| AND | WS08 | 1.14 | 0.02 | 0.03 | 0.00 | 5 | 23.00 | 8.04 | 0.04 | 3.05 | 0.47 | 3.46 | 0.67 | 0.11 | 0.71 |
| AND | WS09 | 1.77 | 0.04 | 0.05 | 0.00 | 6 | 17.00 | 8.90 | 0.03 | 2.67 | 0.17 | 4.69 | 1.35 | 0.19 | 1.31 |
| AND | WS10 | 0.89 | 0.03 | 0.04 | 0.00 | 6 | 32.00 | 8.88 | 0.05 | 2.49 | 0.23 | 4.30 | 1.09 | 0.15 | 1.26 |
| KNZ | edlr | 0.80 | - | - | 0.06 | 16 | - | - | - | - | - | - | - | - | - |
| KNZ | hikx | 0.94 | - | - | 0.29 | 26 | - | - | - | - | - | - | - | - | - |
| LUQ | Q1 | 0.72 | 0.05 | 0.20 | 0.14 | 5.34 | 1.00 | 34.41 | - | 8.58 | 0.91 | 5.52 | 3.11 | 1.28 | 8.35 |
| LUQ | Q2 | 0.85 | 0.05 | 0.19 | 0.13 | 2.50 | 1.00 | 29.48 | - | 7.43 | 0.93 | 5.98 | 2.85 | 0.94 | 7.39 |
| LUQ | Q3 | 0.80 | 0.05 | 0.15 | 0.11 | 2.50 | 1.00 | 28.42 | - | 7.56 | 0.72 | 4.68 | 3.07 | 0.64 | 8.36 |
| LUQ | QG | 0.90 | 0.04 | 0.11 | 0.07 | 2.50 | 1.00 | 15.25 | - | 5.27 | 0.45 | 2.76 | 1.07 | 0.41 | 6.30 |
| LUQ | QP | 1.30 | 0.06 | 0.12 | 0.06 | 2.50 | 1.00 | 19.11 | - | 6.24 | 0.33 | 4.35 | 3.27 | 0.48 | 8.18 |
| LUQ | QS | 1.58 | 0.07 | 0.14 | 0.08 | 2.50 | 1.00 | 11.10 | - | 4.72 | 0.28 | 2.49 | 1.47 | 0.57 | 7.17 |
| LUQ | RI | 0.99 | 0.04 | 0.15 | 0.09 | 7.29 | 1.00 | 19.21 | - | 5.40 | 0.55 | 3.57 | 1.24 | 0.43 | 6.17 |
| LUQ | MPR | 1.12 | 0.06 | 0.13 | 0.06 | 2.50 | 1.00 | 20.84 | - | 6.57 | 0.68 | 7.70 | 1.99 | 1.17 | 7.78 |

**Table S3.** Start and end date of time series for each stream along with the length of the data record. All records started in January and ended in December for every year analyzed for DOC and DON except CPC which started in May and ended in August for every year analyzed. – correspond to no data available**.**

| **Site** | **Stream** | **Start Year** | | **End Year** | | **Total record length** | | |
| --- | --- | --- | --- | --- | --- | --- | --- | --- |
|  |  | **DOC** | **DON** | **DOC** | **DON** | **DOC** | **DON** | |
| LUQ | RI | 2000 | | 2015 | | 15 | | |
| LUQ | MPR | 1998 | | 2015 | | 17 | | |
| LUQ | QP | 2000 | | 2015 | | 15 | | |
| LUQ | Q1 | 1998 | | 2015 | | 17 | | |
| LUQ | Q2 | 1998 | | 2015 | | 17 | | |
| LUQ | Q3 | 1998 | | 2015 | | 17 | | |
| LUQ | QG | 2000 | | 2015 | | 15 | | |
| LUQ | QS | 2000 | | 2015 | | 15 | | |
| AND | MACK | 2003 | | 2015 | | 12 | | |
| AND | WS02 | 2003 | | 2015 | | 12 | | |
| AND | WS08 | 2004 | | 2015 | | 11 | | |
| AND | WS09 | 2004 | | 2015 | | 11 | | |
| AND | WS01 | 2004 | | 2015 | | 11 | | |
| AND | WS06 | 2003 | | 2015 | | 12 | | |
| AND | WS07 | 2003 | | 2015 | | 12 | | |
| AND | WS10 | 2004 | | 2015 | | 11 | | |
| AND | LOOK | 2006 | | 2015 | | 9 | | |
| LMP | CB | 2005 | | 2016 | | 11 | | |
| LMP | LMP73 | 2000 | | 2016 | | 16 | | |
| LMP | LTR | 2004 | | 2016 | | 12 | | |
| LMP | MLB | 2008 | | 2016 | | 8 | | |
| LMP | NBR | 2004 | | 2016 | | 12 | | |
| LMP | NOR | 2004 | | 2016 | | 12 | | |
| LMP | PWT | 2004 | | 2016 | | 12 | | |
| LMP | RMB | 2004 | | 2016 | | 12 | | |
| LMP | WHB | 2003 | | 2016 | | 13 | | |
| FIN | Aur | 1988 | | 2015 | | 27 | | |
| FIN | Eur | 1991 | | 2015 | | 24 | | |
| FIN | Iij | 1995 | | 2015 | | 20 | | |
| FIN | Kal | 1975 | 1984 | 2012 | | 37 | 31 | |
| FIN | Kel | 1989 | 1975 | 2015 | | 26 | 40 | |
| FIN | Kem | 1991 | 1988 | 2015 | | 24 | 27 | |
| FIN | Kii | 1995 | 1975 | 2015 | 2012 | 20 | 37 | |
| FIN | Kis | 1996 | 1989 | 2015 | | 19 | 26 | |
| FIN | Kiv | 1978 | 1991 | 1994 | 2015 | 16 | 24 | |
| FIN | Koj | 1975 | 1995 | 2015 | | 40 | 20 | |
| FIN | Kos | 1994 | 1996 | 2015 | | 21 | 19 | |
| FIN | Kot | 1981 | 1978 | 2001 | 1994 | 20 | 16 | |
| FIN | K.Hur | 1975 | | 2015 | | 40 | | |
| FIN | K.Ahv | 1984 | 1994 | 2015 | | 31 | 21 | |
| FIN | K.Kok | 1988 | 1981 | 2015 | 2001 | 27 | 20 | |
| FIN | Lap | 1996 | | 2012 | 2015 | 16 | 19 | |
| FIN | Les | 1998 | 1995 | 2015 | | 17 | 20 | |
| FIN | Mur | 1978 | | 1994 | | 16 | | |
| FIN | Oul | 1975 | | 2015 | | 40 | | |
| FIN | Paj | 1985 | | 2015 | | 30 | | |
| FIN | Per | 1982 | | 2015 | | 33 | | |
| FIN | Pyh | 1996 | | 2015 | | 19 | | |
| FIN | Sav | 2006 | | 2015 | | 9 | | |
| FIN | Sii | 1983 | | 2015 | | 32 | | |
| FIN | Sim | 1991 | | 2015 | | 24 | | |
| FIN | Ska | 1975 | | 2015 | | 40 | | |
| FIN | Tor | 1991 | | 2015 | | 24 | | |
| FIN | Usk | 1988 | | 2015 | | 27 | | |
| FIN | Val | 1978 | | 1994 | | 16 | | |
| FIN | Vir | 2003 | | 2015 | | 12 | | |
| FIN | Vuo | 1989 | | 2015 | | 26 | | - |
| FIN | Ylij | 1981 | | 2001 | | 20 | - | |
| HBF | W1 | 2006 | | 2015 | | 9 | | |
| HBF | W6 | 1996 | | 2015 | | 19 | | |
| HBF | W7 | 1996 | | 2015 | | 19 | | |
| HBF | W8 | 1996 | | 2015 | | 19 | | |
| HBF | W9 | 1996 | | 2015 | | 19 | | |
| KNZ | edlr | 1994 | | 2016 | | 22 | - | |
| KNZ | hikx | 1994 | | 2016 | | 22 | - | |
| CPC | C1 | 2002 | | 2010 | | 8 | | |
| CPC | C2 | 2002 | | 2010 | | 8 | | |
| CPC | C3 | 2002 | | 2010 | | 8 | | |
| CPC | C4 | 2002 | | 2010 | | 8 | | |
| CPC | CB | 2002 | | 2010 | | 8 | | |
| CPC | CJ | 2002 | | 2010 | | 8 | | |
| CPC | P6 | 2005 | | 2010 | | 5 | | |
| CPC | PC | 2002 | | 2010 | | 8 | | |
| CPC | PJ | 2002 | | 2010 | | 8 | | |

**Table S4.** Sampling rates and analytical methods for analysis of dissolved organic carbon (DOC) or total organic carbon (TOC), total dissolved nitrogen (TDN), total nitrogen (TN) or dissolved organic nitrogen (DON), nitrate (NO_3_^-^), and ammonium (NH_4_^+^) concentrations for each site. – corresponds to no analysis performed.

| **Site** | **Sampling Frequency** | **Sampling and storage** | **TOC or DOC** | **TDN** | **TN or DON** | **NO_3_^-^** | **NH_4_^+^** | **Reference** |
| --- | --- | --- | --- | --- | --- | --- | --- | --- |
| **AND** | ﻿3-week composites | ﻿ Samples composited over a 3-week period, collected weekly and refrigerated; filtered after arriving at the lab and analyzed within 2 days | DOC - combustion | Persulfate digestion and analysis by automated colorimetric,  Technicon Auto-analyzer II | DON - mathematically determined by subtracting NH_3_-N and NO_3_-N from TDN | Colorimetric, automated cadmium reduction | ﻿Colorimetric,  Technicon Auto-analyzer II | Martin and Harr 1998  Johnson et al. 2021 |
| **CPC** | Daily to Bi-weekly | Daily samples were collected with autosamplers as a composite of samples collected at 6 pm and 6 am the following day | DOC - combustion  Shimadzu TOC-5000 | Shimadzu TOC-5000 plumbed to an Antek 7050 nitric oxide detector to quantify total dissolved nitrogen | DON - mathematically determined by subtracting NH_3_-N and NO_3_-N from TDN | Ion chromatography | Ion chromatography | Petrone et al. 2006 |
| **FIN** | Monthly to Weekly | ﻿TOC - deep-frozen and analyzed within 1–3 months  TN - analyzed on the day after sampling | ﻿TOC - oxidized to carbon dioxide by combustion and determined by infrared spectrometry;  DOC - determined as 95% of TOC | ﻿– | TN - analyzed calorimetrically after oxidation with peroxodisulfade and reduction with Cd–Cu column;  DON - determined as 95% of TN | – | – | Mattsson et al. 2005; Kortelainen et al. 2006 |
| **HBF** | Weekly | Since June 1, 2013 samples are brought back to the Pierce Lab, and immediately filtered; Prior, samples were not filtered or frozen before analysis. | DOC - high-temperature catalytic oxidation technique | High-temperature catalytic oxidation technique | DON - mathematically determined by subtracting NH_3_-N and NO_3_-N from TDN | ﻿Ion chromatography | ﻿Colorimetric, automated indophenol- blue method | Buso et al. 2000  Campbell et al. 2021 |
| **LMP** | Weekly | Filtered during collection and frozen until analyzed | ﻿ DOC - high-temperature catalytic oxidation technique | High-temperature catalytic oxidation technique | DON - mathematically determined by subtracting NH_4_-N and NO_3_-N from TDN | ﻿Ion chromatography | ﻿ Colorimetric, SmartChem 200 using the alkaline phenate | Coble et al. 2018  Wymore et al 2021 |
| **KNZ** | Every other day | Frozen until analyzed | DOC - high temperature combustion | – | – | Colorimetric determination on a flow solution analyzer | Colorimetric determination on a flow solution analyzer | Kemp and Dodds 1998 |
| **LUQ** | Weekly | Filtered during collection and frozen until analyzed | ﻿ DOC - high-temperature catalytic oxidation technique | High-temperature catalytic oxidation technique | DON - mathematically determined by subtracting NH_4_-N and NO_3_-N from TDN | Ion chromatography | Colorimetric, SmartChem 200 using the alkaline phenate | Merriam et al. 2002  McDowell et al. 2021 |

**Table S5.** References and repositories for each sites data set

| **Program** | **Dataset** | **URL or DOI** | **Reference if applicable** | **Contact** | **Email** |
| --- | --- | --- | --- | --- | --- |
| LTER | AND  Oregon, USA | https://doi.org/10.6073/pasta/bb935444378d112d9189556fd22a441d | Johnson, S.L and R.L. Fredriksen. 2019. Stream chemistry concentrations and fluxes using proportional sampling in the Andrews Experimental Forest, 1968 to present ver 23. | Sherri Johnson | sherri.johnson2@usda.gov |
| LTER | CPC,  Alaska, USA | https://doi.org/10.6073/pasta/257da36f3edb8df2976696f98a219b7d | Jones, J., F.S. Chapin, R.W. Ruess, and Bonanza Creek LTER. 2014. Stream water chemistry of CPCRW, 2002-2010 ver 20. Environmental Data Initiative. | Jeremy Jones | jay.jones@alaska.edu |
| LTER | HBF  New Hampshire, USA | Submitted by PI or primary contract | NA | Emily Bernhardt | emily.bernhardt@duke.edu |
| LTER | LUQ  Puerto Rico | https://doi.org/10.6073/pasta/f9df56348f510da0113b1e6012fa2967 | -McDowell, W. H. 2017. Chemistry of stream water from the Luquillo Mountains ver 4923051. Environmental Data Initiative.  -W. H. McDowell*,* Leon, C. L., Shattuck, M. D., Potter J. D., Heartsill-Scalley, T., González, G., Shanley, J. B., and Wymore, A. S. 2021. The Luquillo Experimental Forest: catchment science in the montane tropics. Hydrological Processes. | William H. McDowell | bill.mcdowell@unh.edu |
| LTER | KNZ  Kansas, USA | Submitted by PI or primary contract | NA | Walter K. Dodds | wkdodds@ksu.edu |
| Finnish Environment Institute (SYKE) | FIN  Finnish watersheds  (multiple locations) | https://wwwp2.ymparisto.fi/scripts/oiva.asp | NA | Pirkko Kortelainen | pirkko.kortelainen@syke.fi |
| NA | LMP  Lamprey River Hydrological Observatory,  New Hampshire, USA | Submitted by PI or primary contract | Wymore, A.S., M. D. Shattuck, J.D. Potter, L. Snyder, and W. H. McDowell. (2021a). The Lamprey River Hydrological Observatory: suburbanization and changing seasonality. Hydrological Processes. | William H. McDowell | bill.mcdowell@unh.edu |
